# Supplementary material for: Towards unified AI-driven fracture mechanics: the extended deep energy method (XDEM)
Source: Nat Commun. 2026 Aug 15;17:8492. doi: 10.1038/s41467-026-76748-1 (PMC13484471; doi:10.1038/s41467-026-76748-1)
Supplement: Supplementary file 3 [file 41467_2026_76748_MOESM3_ESM.zip › pic/Results/Crack_propagation/Straight/2D_fdGraph.pdf]

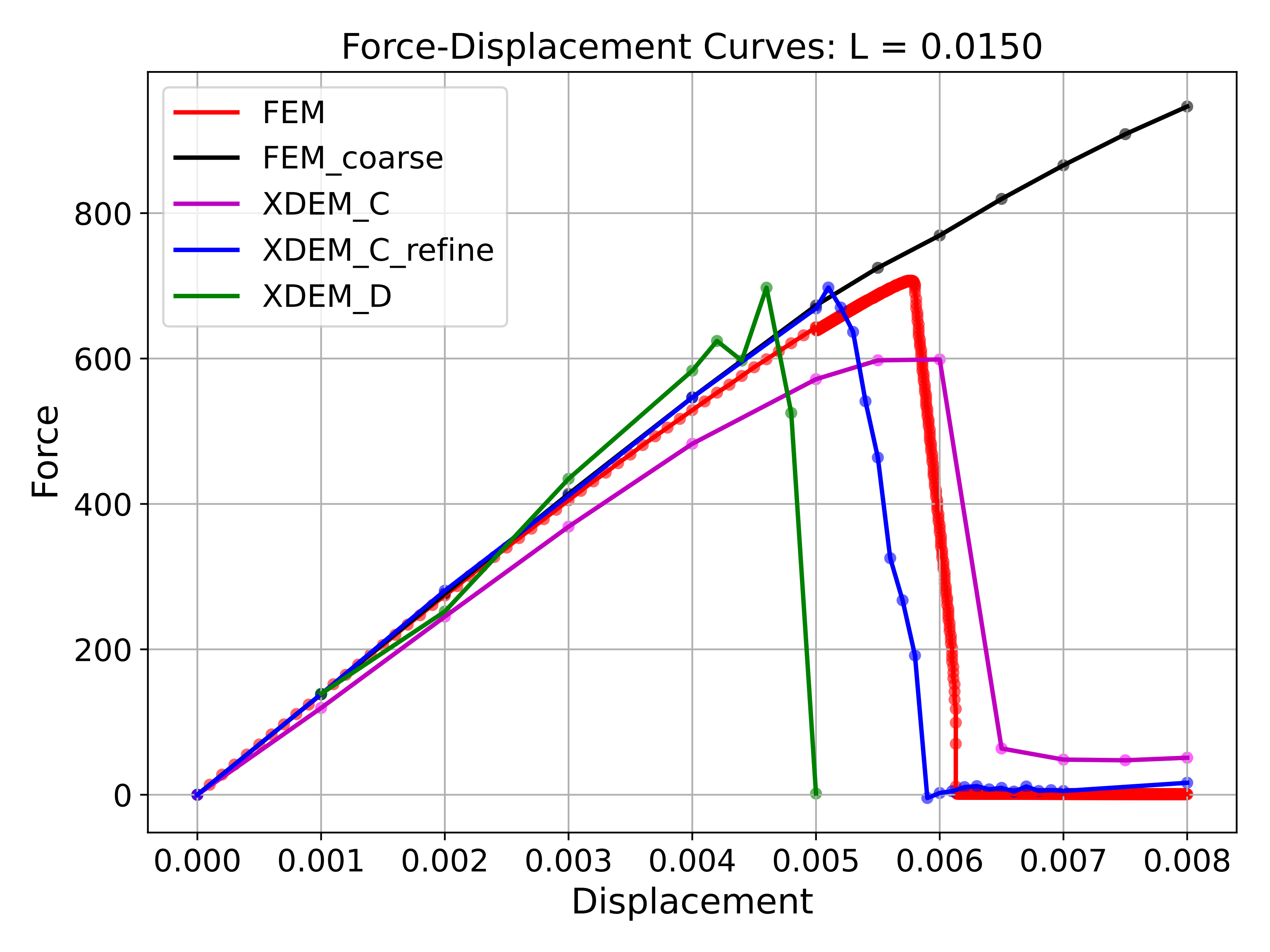

Force-Displacement Curves: L = 0.0150
FEM
FEM_coarse
XDEM_C
XDEM_C_refine
XDEM_D
800
600
400
200
0
Force
0.000
0.001
0.002
0.003
0.004
0.005
0.006
0.007
0.008
Displacement
